# Unique Toll-Like Receptor 4 Activation by NAMPT/PBEF Induces NFκB Signaling and Inflammatory Lung Injury

**DOI:** 10.1038/srep13135

**Published:** 2015-08-14

**Authors:** Sara M. Camp, Ermelinda Ceco, Carrie L. Evenoski, Sergei M. Danilov, Tong Zhou, Eddie T. Chiang, Liliana Moreno-Vinasco, Brandon Mapes, Jieling Zhao, Gamze Gursoy, Mary E. Brown, Djanybek M. Adyshev, Shahid S. Siddiqui, Hector Quijada, Saad Sammani, Eleftheria Letsiou, Laleh Saadat, Mohammed Yousef, Ting Wang, Jie Liang, Joe G. N. Garcia

**Affiliations:** 1 Department of Medicine and Arizona Respiratory Center, The University of Arizona; 2 Institute for Personalized Respiratory Medicine, University of Illinois at Chicago; 3 Department of Anesthesiology, University of Illinois at Chicago; 4 Department of Bioengineering, University of Illinois at Chicago; 5 Life Science Group, Bio-Rad Laboratories, Inc.

## Abstract

Ventilator-induced inflammatory lung injury (VILI) is mechanistically linked to increased *NAMPT* transcription and circulating levels of nicotinamide phosphoribosyl-transferase (NAMPT/PBEF). Although VILI severity is attenuated by reduced NAMPT/PBEF bioavailability, the precise contribution of NAMPT/PBEF and excessive mechanical stress to VILI pathobiology is unknown. We now report that NAMPT/PBEF induces lung NFκB transcriptional activities and inflammatory injury via direct ligation of Toll–like receptor 4 (TLR4). Computational analysis demonstrated that NAMPT/PBEF and MD-2, a TLR4-binding protein essential for LPS-induced TLR4 activation, share ~30% sequence identity and exhibit striking structural similarity in loop regions critical for MD-2-TLR4 binding. Unlike MD-2, whose TLR4 binding alone is insufficient to initiate TLR4 signaling, NAMPT/PBEF alone produces robust TLR4 activation, likely via a protruding region of NAMPT/PBEF (S402-N412) with structural similarity to LPS. The identification of this unique mode of TLR4 activation by NAMPT/PBEF advances the understanding of innate immunity responses as well as the untoward events associated with mechanical stress-induced lung inflammation.

Mechanical ventilation is life-saving in critically ill patients experiencing respiratory failure as a result of acute respiratory distress syndrome (ARDS), an inflammatory lung syndrome with considerable morbidity and mortality^1^,^2^,^3^. Unfortunately, mechanical ventilation delivered to injured lungs also results in excessive mechanical stress that directly contributes to the magnitude of lung injury and severity of ARDS, a process known as ventilator–induced lung injury or VILI^3^,^4^. VILI shares many ARDS pathologic features such as marked increases in lung vascular leakage, inflammatory cell influx, and inflammatory cytokine expression^4^,^5^. The pathobiologic mechanisms underlying VILI and ARDS, however, remain unclear and effective pharmacotherapies have yet to emerge.

Our prior genomic–intensive approaches in multi-species preclinical models of ARDS and VILI^6^,^7^,^8^,^9^ identified *NAMPT*, a highly novel candidate gene^9^,^10^ encoding nicotinamide phosphoribosyl-transferase (NAMPT). NAMPT is also known as pre-B cell colony-enhancing factor (PBEF) or visfatin, and plasma levels of NAMPT/PBEF serve as a biomarker in humans with ARDS^10^. *NAMPT* gene variants alter promoter activity to increase NAMPT/PBEF expression and confer significantly increased susceptibility and mortality to ARDS^10^,^11^. In preclinical models of VILI, NAMPT/PBEF expression was spatially localized to lung epithelium, tissue leukocytes and the lung vascular endothelium^10^ with direct participation in ARDS/VILI pathobiology. Furthermore, intra-tracheally-instilled NAMPT/PBEF induces a neutrophilic alveolitis^12^ and heterozygous PBEF^+/−^ mice are dramatically protected from severe murine VILI^12^. As reductions in extracellular NAMPT/PBEF availability, via neutralizing antibodies^12^ or liposomes encargoed with *NAMPT* siRNAs, provide significant protection from LPS- and VILI-induced murine lung inflammation^12^, together these findings indicate that NAMPT/PBEF is an attractive therapeutic target in ARDS and VILI.

NAMPT regulates intracellular nicotinamide adenine dinucleotide (NAD) biosynthesis and apoptosis pathways^11^,^13^,^14^,^15^. However, it is the increased NAMPT/PBEF expression and extracellular secretion into blood and bronchoalveolar lavage fluid that produce the profound inflammatory effects of NAMPT/PBEF in response to inflammatory stimuli such as excessive mechanical stress^10^,^12^. In the absence of an inflammatory stimulus such as LPS, recombinant PBEF alone directly exerts inflammatory responses that are similar to the ARDS condition^12^. Contributing to potential mechanisms of NAMPT/PBEF-mediated lung pathobiology, we demonstrated that exogenous NAMPT/PBEF elicits robust inflammatory gene transcription in murine lungs^12^, including dysregulated genes in the transcriptome related to leukocyte extravasation, the transcription factor NFκB^12^, and expression of Toll-like receptors (TLR)^12^,^16^.

These data, supporting NAMPT/PBEF as a regulator of lung innate immunity pathways, led us to systematically explore the biochemical and molecular basis for NAMPT/PBEF involvement in the inflammatory pathophysiology associated with mechanical ventilation and acute lung injury. Utilizing complementary system biology *in vivo* and *in vitro* approaches, including genetically-engineered mice and computational modeling, we now define novel and rapid NAMPT/PBEF-mediated NFκB transcriptional activities via the ligation of TLR4. Computational analysis revealed substantial sequence identity between NAMPT/PBEF and MD-2, a TLR4-binding protein essential for LPS-induced TLR4 activation. Importantly, NAMPT/PBEF and MD-2 share similar α helix and β sheet structures and strong similarity in regions containing the majority of MD-2-TLR4 binding residues. We further speculate that a protruding region of NAMPT/PBEF (S402-N412), with structural similarity to LPS, serves as the site of direct TLR4 binding. Wheras MD-2 binding of TLR4 in the absence of LPS fails to induce NFκB activation, our identification of a novel mechanism of direct TLR4 activation by NAMPT/PBEF, occurring in the absence of bacterial infection and cofactor requirements, increases the understanding of lung innate immunity responses and the untoward inflammatory effects of mechanical stress–induced lung injury.

## Results

### Exogenous NAMPT/PBEF induces robust *in vitro* and *in vivo* NFκB activation in human and murine tissues

**Figure 1:**
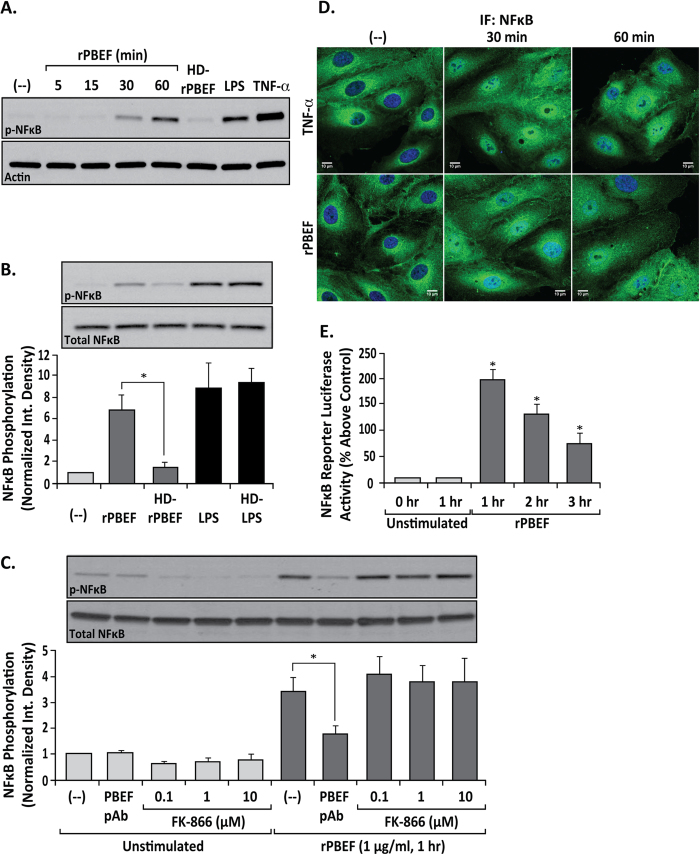
Extracellular NAMPT/PBEF independently mediates rapid NFκB activation in human lung endothelium. (**Panel A**) Activation of NFκB signaling in human lung EC challenged with recombinant NAMPT/PBEF (rPBEF) (1 μg/ml, 5–60 min). Stimulated EC lysates were probed for p-NFκB (Ser^536^) or control β-actin. Heat-denatured rPBEF (HD-rPBEF) (1 μg/ml, 1 hr) served as a negative control confirming that rPBEF effects do not reflect endotoxin contamination. LPS (5 μg/ml, 1 hr) and TNF-α (100 ng/ml, 15 min) served as positive controls for NFκB signaling pathway activation. n = 3; representative blots shown. (**Panel B**) NAMPT/PBEF-mediated NFκB activation does not reflect endotoxin contamination. rPBEF or LPS were exposed to 100 °C for 5 min. Human lung ECs were treated with rPBEF (1 μg/ml), heat denatured (HD)-rPBEF (1 μg/ml), LPS (5 μg/ml), or HD-LPS (5 μg/ml) for 1 hr, EC lysates were then probed for p-NFκB (Ser^536^) or total NFκB. (**Panel C**) NAMPT/PBEF enzymatic inhibitor FK-866 fails to attenuate rPBEF-induced NFκB phosphorylation. Human lung ECs were treated with rPBEF (1 μg/ml, 1 hr) either without any pretreatment, with premixing with neutralizing NAMPT/PBEF pAb (100 μg/ml, 30 min), or pretreatment with FK-866 (0.1–10 μM, 1 hr). EC lysates were probed for p-NFκB (Ser^536^) and total NFκB. For (**Panel B,C**) bar graphs represent data as integrated density normalized to unstimulated control. n = 3–9 (**B**) or n = 4–6 (**C**); *p < 0.01 (**B**) or *p < 0.05 (**C**) vs rPBEF-stimulated control. (**Panel D**) Immunofluorescent monitoring of agonist-induced NFκB translocation to the nucleus. rPBEF (10 μg/ml) for 0–60 min induces NFκB translocation from the cytosol to the nuclei in human lung microvascular EC similar to EC challenge with TNF-α (100 ng/ml). Scale bar = 10 μm; n = 3; representative immunofluorescence images shown. (**Panel E**) rPBEF (10 ng/mL)-mediated increases in NFκB promoter-driven luciferase reporter activity in human lung EC assessed from 0–3 hr. NFκB promoter activity following rPBEF challenge was measured using a dual luciferase reporter assay and absorbance of the inducible NFκB promoter-responsive firefly luciferase construct was normalized to the absorbance of the constitutively active Renilla luciferase construct (internal control). Bar graphs represent data as percentage luciferase activity increase above control, n = 3; *p < 0.05 versus unstimulated controls.

Leveraging our prior reports of NFκB transcriptome induction by recombinant NAMPT/PBEF (rPBEF)^12^,^17^,^18^, complementary *in vitro* approaches were utilized to functionally examine the direct role of extracellular NAMPT/PBEF in NFκB pathway activation and innate immunity gene expression. Initial experiments assessed exogenous NAMPT/PBEF-mediated phosphorylation of NFκB (p-NFκB at Ser^536^) in human lung endothelial cells (EC) as an indication of NFκB activation. rPBEF, similar to LPS and TNF-α, increases phosphorylation of p-NFκB within 30 min with persistent elevation at 1 hr (Fig. 1A). Heat denaturing of rPBEF (HD-rPBEF) resulted in the elimination of NAMPT/PBEF’s capacity to induce NFκB activation, demonstrating that NAMPT/PBEF-mediated NFκB phosphorylation/activation does not reflect endotoxin contamination (Fig. 1A,B). rPBEF-induced NFκB activation was unaffected by inhibition of NAMPT enzymatic activity (FK-866), indicating that phosphoribosyl-transferase activity is not required (Fig. 1C).

Time-dependent NFκB translocation from the cytoplasm to the nucleus was next assessed as a reflection of activation of the canonical NFκB pathway. Consistent with biochemical indices of NFκB activation, rPBEF challenge resulted in NFκB translocation to the nucleus in human lung EC (Fig. 1D) temporally similar to TNF-α (positive control) (Fig. 1D). Finally, EC transfected with a NFκB promoter luciferase reporter and challenged with rPBEF exhibited increased luciferase activity (up to three hours) (Fig. 1E). These data strongly suggest that extracellular NAMPT/PBEF is a rapid and potent NFκB activator in human lung endothelium.

**Figure 2:**
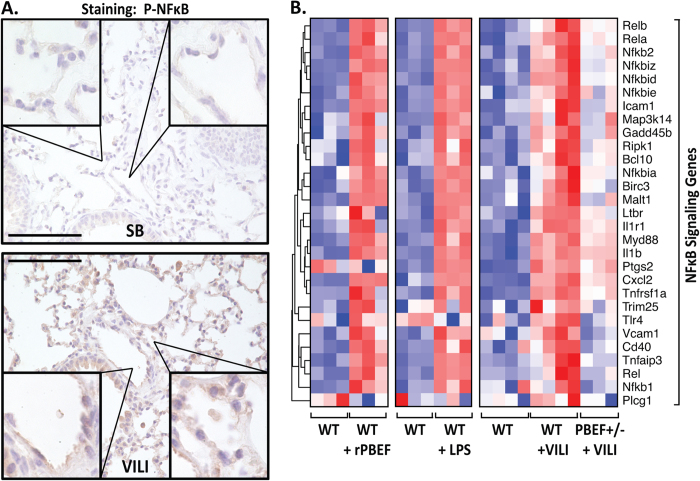
Extracellular NAMPT/PBEF mediates rapid NFκB activation in murine lungs. (**Panel A**) Paraffin-embedded lung tissue was sectioned (10 μm) and immunostained with p-NFκB antibody. Shown are representative images where prominent NFκB activation is evident in VILI-challenged mice, compared to spontaneously breathing (SB) mice, with prominent p-NFκB expression in capillary endothelium and alveolar epithelium. Scale bar = 100 μm. See Supplemental Figure 1 for IHC staining isotype controls. (**Panel B**) Heat maps reflecting the highly upregulated expression of NFκB pathway genes in response to rPBEF (40 μg/mouse, 4.5 hr), LPS (2.5 mg/kg, 4 hr), and VILI challenge (30 ml/kg tidal volume, 4 hr). VILI-mediated increases in NFκB pathway gene expression were markedly reduced in heterozygous PBEF^+/−^ mice. Blue color indicates reduced gene expression, red color reflects increased gene expression.

Extracellular NAMPT/PBEF-mediated NFκB activation was next evaluated in preclinical models of ARDS/VILI. Phospho-NFκB immunostaining of murine lung tissues from VILI-exposed mice revealed prominent VILI-induced NFκB phosphorylation/activation in vascular endothelium and alveolar epithelium (Fig. 2A). Genome–wide gene expression analysis in wild type mice revealed marked similarities in NFκB Toll receptor pathway signaling gene upregulation evoked by rPBEF, LPS, and VILI, with heterozygous PBEF^+/−^ mice exhibiting dramatic attenuation of VILI-mediated NFκB pathway gene expression (Fig. 2B). These studies provide compelling evidence for extracellular NAMPT/PBEF involvement in induction of the NFκB transcriptome and in lung innate immunity directly contributing to ARDS/VILI pathobiology.

### NAMPT/PBEF is a novel TLR4 agonist triggering NFκB activation

**Figure 3:**
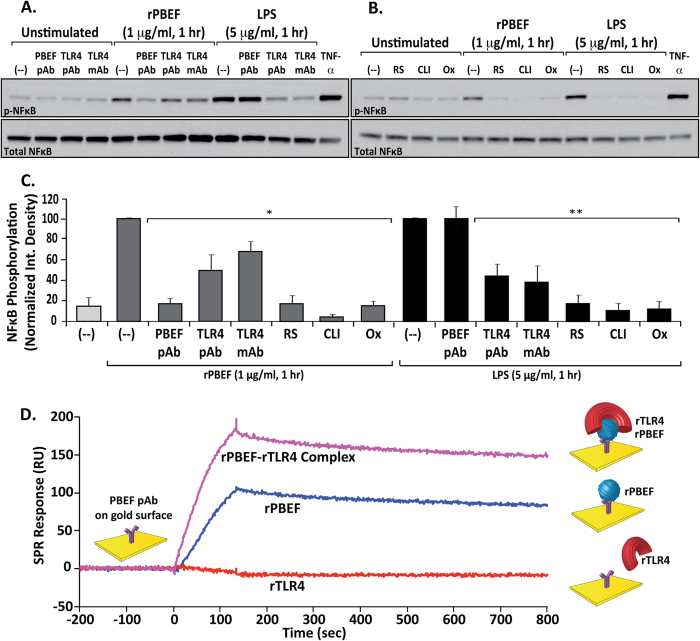
Extracellular NAMPT/PBEF induces NFκB activation via TLR4 ligation *in vitro*. **Panels A and B**. Effects of TLR4 inhibitory strategies on rPBEF- and LPS-mediated NFκB phosphorylation in ECs. Cell lysates were immunoblotted for phospho-specific or total NFκB with experiments independently performed in triplicate (representative blots shown). TNF-α (100 ng/ml, 15 min) serves as a positive control for NFκB signaling activation. (**Panel A**) Inhibition of both rPBEF (1 μg/ml, 1 hr)- and LPS (5 μg/ml, 1 hr)-mediated NFκB phosphorylation (Ser^536^) in EC pretreated (1 hr) with neutralizing TLR4 polyclonal (pAb, 20 μg/ml) or TLR4 monoclonal antibodies (mAb, 10 μg/ml). Premixing of rPBEF, but not LPS, with neutralizing NAMPT/PBEF pAb (100 μg/ml, 30 min) similarly reduced NFκB phosphorylation. (**Panel B**) Inhibitory effects of 1 hr pretreatment with TLR4 pharmacologic inhibitors (RS-LPS [10 μg/ml], CLI-095 [5 μM] and OxPAPC [30 μg/ml]) on rPBEF- and LPS-mediated NFκB phosphorylation in ECs. (**Panel C**) Densitometric summary of the attenuation of rPBEF- and LPS-induced NFκB phosphorylation by TLR4 neutralizing antibodies and inhibitors. rPBEF-induced NFκB phosphorylation was significantly reduced to levels similar to those observed with pretreatment with anti-NAMPT/PBEF specific antibody. LPS-induced NFκB phosphorylation was unaffected by pretreatment with anti-NAMPT/PBEF specific antibody. Bar graphs represent data as integrated density normalized to rPBEF- or LPS-stimulated control. n = 3 independent experiments per condition; *p < 0.05 versus rPBEF-stimulated control, **p < 0.05 versus LPS-stimulated control. Pretreatment with inhibitors or neutralizing antibodies alone (without rPBEF or LPS stimulation) did not significantly differ from unstimulated controls (data not shown). (**Panel D**) Surface plasmon resonance (SPR) analysis (using Bio-Rad ProteOn XPR36 instrument and GLC sensor chips) demonstrates TLR4-NAMPT/PBEF binding interaction. Bethyl PBEF antibody was covalently bound to the chip surface, using standard direct immobilization, at a final immobilization level of 5000 RUs. rPBEF only (100 nM), rTLR4 only (1 μM), and rPBEF-rTLR4 pre-mixed (100 nM or 1 μM, respectively) analytes were then injected over the PBEF antibody coated surface. While rTLR4 did not bind to the PBEF antibody coated surface, pre-mixed rPBEF-rTLR4 resulted in increased binding response over rPBEF alone.

Given the prominent NFκB and TLR pathway gene signatures evoked by NAMPT/PBEF, we assessed TLR4 as a putative NAMPT/PBEF receptor. Human lung EC pretreated with either TLR4 antibodies (10–20 μg/ml, 1 hr) or TLR4 inhibitors (RS-LPS 10 μg/ml, CLI-095 5 μM, OxPAPC 30 μg/ml; 1 hr), were challenged with rPBEF or LPS and cell lysates immunoblotted for either phosphorylated or total NFκB. In each case, inhibition of TLR4 activity resulted in marked reductions in rPBEF-mediated Ser^536^ NFκB phosphorylation (Fig. 3A–C). The biologic activity of rPBEF was confirmed by NAMPT/PBEF-specific antibody inhibition (Fig. 3A,C). TLR4 inhibition also reduced LPS-mediated Ser^536^ NFκB phosphorylation. In contrast to rPBEF, however, LPS-mediated Ser^536^ NFκB phosphorylation was unaffected by a NAMPT/PBEF neutralizing antibody (Fig. 3A,C), strongly supporting TLR4 as a novel NAMPT/PBEF receptor. Surface plasmon resonance (SPR) analysis was next utilized to demonstrate direct molecular interaction of rTLR4 and rPBEF. Recombinant TLR4 failed to bind to the PBEF antibody coated surface whereas a previously mixed solution of rPBEF and rTLR4 resulted in strong increases in binding over rPBEF alone (Fig. 3D).

**Figure 4:**
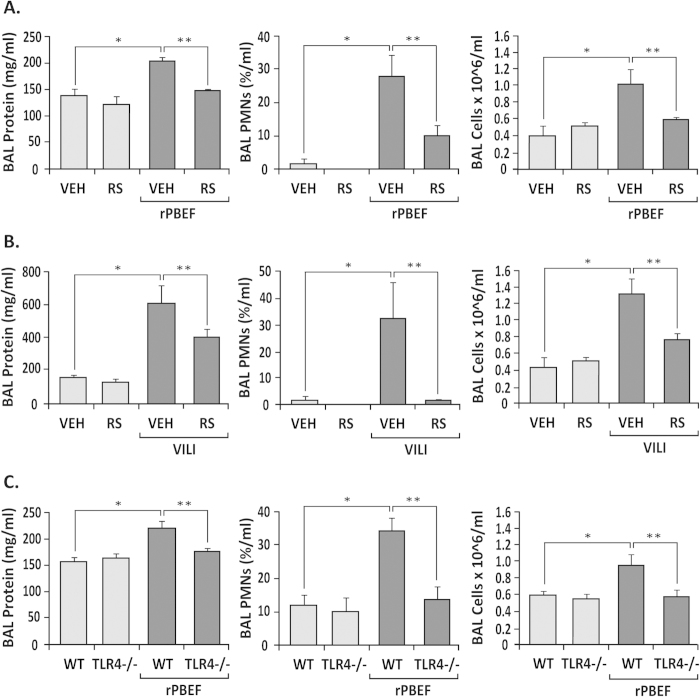
TLR4 is the novel receptor for extracellular NAMPT/PBEF- and VILI-induced NFκB activation and inflammatory lung injury *in vivo*. Panel A. Compared to saline-challenged controls (VEH), intratracheal instillation of rPBEF (40 μg/mouse) in wild type mice produces robust increases in BAL protein levels, in the percentage of BAL PMNs, and in BAL total cell counts, findings consistent with prior reports^12^. These rPBEF–mediated inflammatory lung indices were significantly reduced both in mice pretreated with the TLR4 inhibitor RS-LPS (100 μg/mouse, i.p.) (**Panel A**) and in TLR4^−/−^ mice (**Panel C**) indicating that TLR4 is required for NAMPT/PBEF-induced pro-inflammatory activities and lung injury. Results are expressed as mean ± SEM; n = 3–6 per condition; *p < 0.05 for VEH/WT vs VEH/WT + rPBEF and **p < 0.05 VEH/WT + rPBEF vs RS/TLR4^−/−^ + rPBEF, using Anova non-parametric Newman-Keuls Multiple Comparison Test. (**Panel B**) Exposure to VILI (40 ml/kg, 4 hr) in wild type mice produces significant increases in BAL protein levels, in the percentage of BAL PMNs, and in BAL cell counts. These rPBEF–mediated inflammatory indices were significantly reduced in mice pretreated with the TLR4 inhibitor RS-LPS (100 μg/mouse), indicating that TLR4 is required for NAMPT/PBEF-induced pro-inflammatory activities and lung injury. Results are expressed as mean ± SEM; n = 3–6 per condition; *p < 0.05 for VEH vs VEH + VILI and **p < 0.05 VEH + VILI vs RS + VILI.

**Figure 5:**
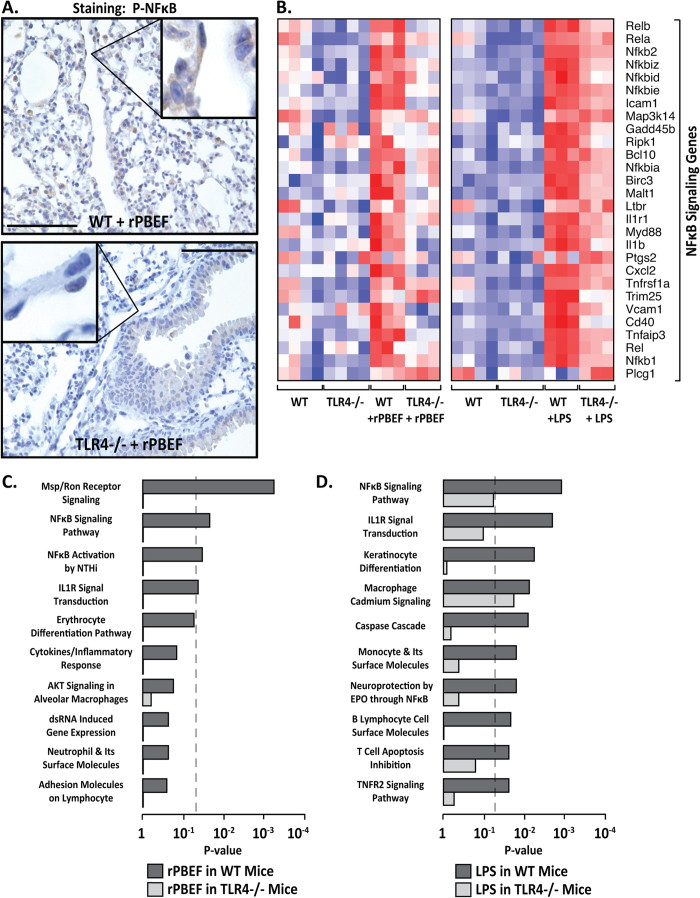
Extracellular NAMPT/PBEF- and LPS-induced NFκB pathway gene dysregulation is mediated by TLR4. (**Panel A**) Paraffin-embedded lung tissue was sectioned (10 μm) and immunostained with a p-NFκB antibody. Shown is a representative image demonstrating prominent NFκB activation and expression in capillary endothelium and alveolar epithelium from rPBEF-challenged wild type mice, whereas p-NFκB immunoreactivity was significantly reduced in rPBEF-challenged TLR4^−/−^ mice. Scale bar = 100 μm. See Supplemental Figure 1 for IHC staining isotype controls. (**Panel B**) Heat maps reflecting the critical involvement of TLR4 in rPBEF- and LPS-mediated upregulation of NFκB pathway gene expression. Both rPBEF (40 μg/mouse, 4.5 hr) and LPS (2.5 mg/kg, 4 hr) mediate robust NFκB pathway increases in wild type mice whereas this expression was markedly reduced in TLR4^−/−^ mice. Blue color indicates reduced gene expression, red color reflects increased gene expression. Bar graphs represent enriched pathways in mice challenged with rPBEF (40 μg/mouse, 4.5 hr) (**Panel C**) or LPS (2.5 mg/kg, 4 hr) (**Panel D**). The top ranking BIOCARTA pathways are listed for the genes differentially expressed between controls and wild-type mice challenged with rPBEF and LPS, respectively. The corresponding pathway patterns for the genes differentially expressed between the wild-type controls and TLR4^−/−^ mice treated with rPBEF or LPS are also indicated. The genes dysregulated by LPS were identified using criteria of a false discovery rate (FDR) of <5% and a minimum of a 2-fold change. The genes dysregulated by rPBEF were identified by a cutoff of <10% FDR and >1.5-fold change. The gray dash line indicates the cutoff of significance (*P*-value < 0.05).

These *in vitro* results were extended to *in vivo* studies utilizing mice pretreated with the TLR4 inhibitor, RS-LPS, as well as TLR4^−/−^ mice (Fig. 4). Consistent with our prior report^12^, rPBEF instillation produced marked increases in lung inflammatory indices in wild type mice (BAL protein levels, BAL PMNs, and BAL cell counts (Fig. 4A)) that were significantly attenuated by RS-LPS pretreatment (100 μg/mouse) (Fig. 4A) in wild type mice. Similar to NAMPT/PBEF challenge, RS-LPS pretreatment produced attenuation of VILI-induced pulmonary inflammation in wild-type mice (Fig. 4B). rPBEF-induced lung inflammation was also reduced in TLR4^−/−^ mice compared to wild type mice (Fig. 4C). TLR4^−/−^ mice demonstrated abolishment of the prominent rPBEF-induced NFκB phosphorylation in murine pulmonary EC (Fig. 5A) and reduced basal levels of NFκB signaling (Fig. 5B). Both rPBEF and LPS triggered similar, robust increases in expression of NFκB signaling genes in wild type animals that were markedly reduced in TLR4^−/−^ mice (Fig. 5B). More importantly, the NFκB pathways gene ontology was significantly dysregulated by either rPBEF or LPS (as the top regulated pathways) with significant suppression of gene dysregulation in TLR4^−/−^ mice (Fig. 5C,D). These results are consistent with the requirement for TLR4 participation in NAMPT/PBEF-induced pro-inflammatory activities and lung injury.

### *In silico* modeling reveals NAMPT/PBEF binding surface similarities with MD-2, an essential LPS cofactor in TLR4 activation

**Figure 6:**
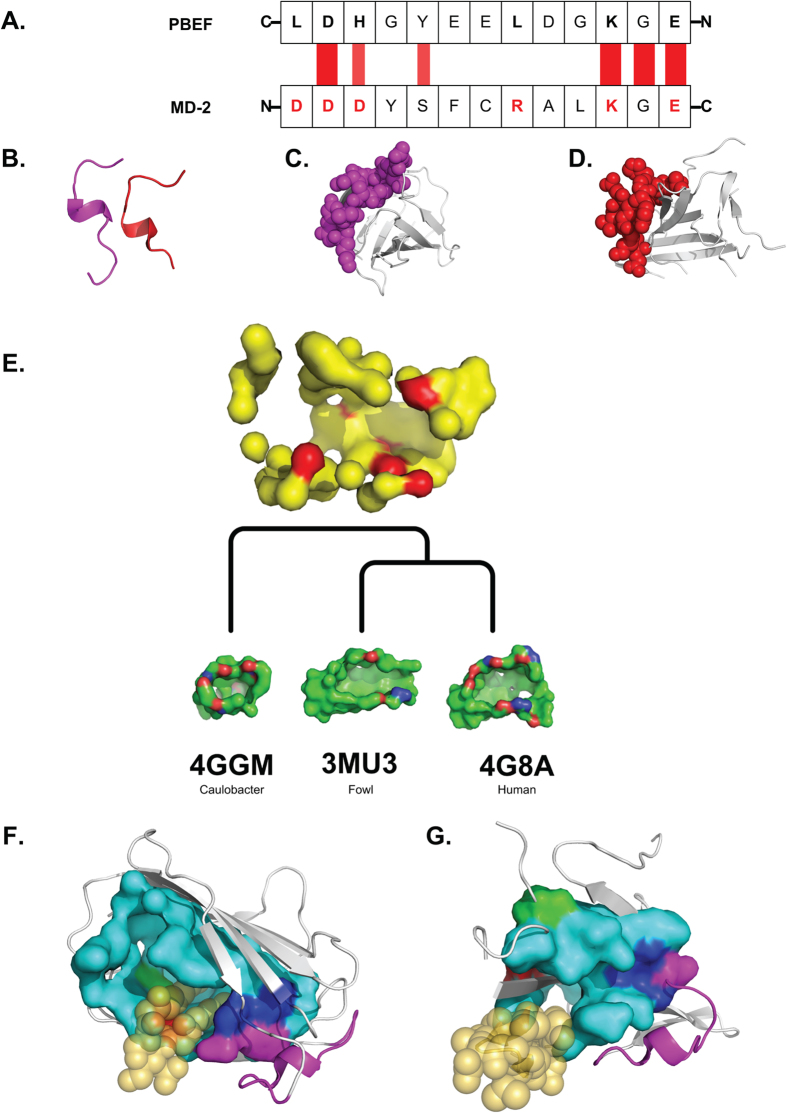
*In silico* modeling of NAMPT/PBEF and MD-2 interactions with TLR4. (**Panel A**) Murine sequence of TLR4-binding loop from MD-2 in the N-to-C order aligned with the loop from murine NAMPT/PBEF in the reverse C-to-N order. Residues in red are known to be involved in MD-2 binding to TLR4^20^. The wider red bands indicate aligned identical residues, while the narrower bands indicate aligned residues with similar physicochemical properties. (**Panel B**) Corresponding structural alignment between the loop in MD-2 (purple) and the loop in NAMPT/PBEF (red). (**Panel C**) Structure of MD-2, where the surface of the TLR4-binding loop of MD-2 (99D-111E) is in purple. (**Panel D**) Structure of NAMPT/PBEF, where the surface of the aligned binding loop from NAMPT/PBEF (457L-445E) is in red. (**Panel E**) Signature pocket for the LPS-binding surface constructed from three diverse LPS binding proteins, each less than 64% of sequence identities with MD-2 but all exhibiting the same LPS binding mode as that of MD-2. Binding pockets of the three LPS binding proteins (red = oxygen; blue = nitrogen; green = carbon atoms) are shown along the hierarchical tree based on sequence order independent structural alignment of the binding surfaces^28^. The large yellow pocket at the root of the tree is the overall signature pocket constructed from the 7 LPS binding pockets. Spatially preserved signature LPS binding residues are in red. (**Panel F**) Signature residues in the LPS binding pocket of the MD-2-LPS complex including F119 (red); I52 (green); L74, L94 (blue); overall LPS binding pocket (cyan); TLR4-binding loop (purple). (**Panel G**) NAMPT/PBEF structure with residues corresponding to signature residues for LPS binding mapped in MD-2: F399 corresponding to F119 on MD-2 (red); L432 and L458 corresponding to L74 and L94, respectively (blue); I114 corresponding to I52 (green); other residues of LPS binding pocket (cyan); loop residues for TLR4-binding loop (purple). Signature residues for LPS binding exhibit greater spatial separation in NAMPT/PBEF compared to MD-2 with the prominent surface pocket for LPS binding, present in MD-2, absent in NAMPT/PBEF. Potential TLR4 activation by the LPS molecule (transparent-wheat) can be mapped to the protruding region of S402-N412 residues on NAMPT/PBEF (transparent-wheat).

The crystal structure of NAMPT/PBEF^19^, the TLR4 receptor, as well as the LPS- and TLR4-binding protein, MD-2, have been resolved^20^,^21^. *In silico* protein structure analysis revealed that despite only ~30% total sequence identity between murine NAMPT/PBEF and MD-2, striking structural similarities exist between a loop region on NAMPT/PBEF and a loop region on MD-2 known to be involved in LPS binding to TLR4 and critical to subsequent TLR4 activation (Fig. 6A,B). Using a sequence order independent structural alignment method^22^, a loop (99D-111E, purple Fig. 6C) in the TLR4-binding region on MD-2 was well aligned in the N-to-C order with a loop residing in NAMPT/PBEF in the reverse C-to-N order (457L-445E, red, Fig. 6D, 30.3% identity). Six of the 7 MD-2 residues known to be important for TLR4 binding reside within this loop^20^ and this loop also contains a prominent motif consisting of a consecutive triplet of residues K109, G110, and E111, a D residue and an S/Y residue^23^ (Fig. 6A) fully conserved among different species. This motif is also present in the NAMPT/PBEF loop (thus indicating a potentially conserved biological function). We speculate that this loop represents the TLR4-binding region for NAMPT/PBEF is supported by protein analysis of the TLR4-binding protein, Der-p2, a house mite allergen with MD-2 homology (sequence identity ~26%)^24^,^25^,^26^. The TLR4-binding regions of Der-p2 are highly conserved and similarly align to the loop in NAMPT/PBEF. Finally, residue R434, in spatial proximity of the predicted NAMPT/PBEF loop binding residues, is structurally aligned to R90 on MD-2 implicated to participate in TLR4-MD-2 interactions^27^. Thus, the observed similarity in the TLR4 binding environment between NAMPT/PBEF and MD-2, in conjunction with the biochemical and SPR studies, support our hypothesis that NAMPT/PBEF is a TLR4-binding protein.

We next constructed a surface model of signature binding site pockets^22^,^28^ characterizing LPS-binding regions based on three structures from species-diverse LPS-binding proteins sharing less than 64% sequence identities with MD-2 (Fig. 6E) but that exhibited identical LPS binding modes as MD-2. According to the degree of residue preservation in their geometric locations in the calculated signature LPS binding surface, the most important signature residues on MD-2 for LPS binding were identified as F119, L74, L94, and I52 (Fig. 6E), residues within the three proteins used to construct the signature pocket. These signature LPS-binding residues, while tightly clustered in MD-2, in NAMPT/PBEF are spatially separated from each other suggesting that NAMPT/PBEF is unlikely to directly bind LPS. For example, the distance between LPS-binding residues F119 (**red in** Fig. 6F) and I52 (**green in** Fig. 6F) in MD-2 is 3.8 Å, whereas the distance between the corresponding residues F399 (**red in** Fig. 6G) and I114 (**green in** Fig. 6G) in NAMPT/PBEF is 9.3 Å. Furthermore, although a well-defined surface pocket exists in MD-2 for LPS binding, no such surface pocket exists on NAMPT/PBEF to contain the LPS molecule. Thus, it is unlikely that NAMPT/PBEF directly interacts with either LPS or MD-2 physically at this region. Interestingly, within the structure of the MD-2-LPS complex, the LPS molecule maps to the protruding region of NAMPT/PBEF (S402-N412) (Fig. 6G), suggesting that NAMPT/PBEF may have intrinsically adopted a conformation capable of directly binding and activating TLR-4. Future mutation studies of these residues will provide further insight into structure–function relationship of NAMPT/PBEF-induced TLR4 signaling.

## Discussion

Originally named for facilitation of B cell maturation, NAMPT/PBEF is a “cytozyme” dually functioning as an intracellular dimeric type II nicotinamide phosphoribosyltransferase enzyme (NAMPT) involved in NAD biosynthesis^29^,^30^ and as an extracellular pro-inflammatory cytokine^12^. NAMPT/PBEF expression is markedly elevated in acutely inflamed lungs in ARDS and VILI^10^,^12^, in cardiac tissues during cardiac arrest and resuscitation^31^, in amniotic membranes during gestation^32^ and is released from visceral fat during the development of obesity, an observation resulting in its naming as visfatin^9^. We recently demonstrated that *NAMPT* expression in the lung is transcriptionally regulated by excessive mechanical stress via STAT5-dependent increases in *NAMPT* promoter activity^10^,^33^ and via post-transcriptional epigenetic mechanisms involving 5′ UTR promoter demethylation and 3′ UTR miRNA binding^33^,^34^. Furthermore, *NAMPT* expression is influenced by promoter SNPs that function to increase NAMPT/PBEF expression and confer enhanced susceptibility to ARDS as well as increased ARDS mortality^10^,^11^,^33^.

We previously explored mechanisms of NAMPT/PBEF-mediated inflammatory lung injury relevant to both ARDS and VILI utilizing multiple complementary *in vitro* and *in vivo* approaches and demonstrated that extracellular NAMPT/PBEF is a direct neutrophil chemoattractant augmenting lung injury induced by excessive mechanical stress (VILI) and inducing robust inflammatory cytokine expression^10^,^12^,^35^. Furthermore, reductions in NAMPT/PBEF bioavailability (neutralizing antibodies or siRNAs) attenuated VILI-induced lung inflammation^12^. The current study expands support for *NAMPT/*PBEF as a novel ARDS/VILI candidate gene and biomarker directly involved in ARDS/VILI pathobiology^10^,^12^. We have now detailed robust NAMPT/PBEF-mediated NFκB activation that is independent of NAMPT enzymatic activity (Fig. 1C). Lung gene ontology signatures evoked by extracellular NAMPT/PBEF exposure demonstrated prominent overlap with Toll-like receptor signaling^12^ and led to interrogation of TLR4 as a putative NAMPT/PBEF receptor. This hypothesis was confirmed by studies utilizing TLR4 pharmacologic inhibitors, TLR4 neutralizing antibodies, TLR4 siRNAs, NAMPT/PBEF–TLR4 SPR analysis, and TLR4^−/−^ mice. TLR4 is unequivocally required for NAMPT/PBEF-mediated pro-inflammatory activities in the lung and presumably in other tissues^31^,^36^,^37^,^38^.

Toll-like receptors (TLRs) are essential to innate immunity responses^39^,^40^ and have been implicated in the pathobiology of acute inflammatory lung injury^41^. Similar to other TLRs, TLR4 recognizes conserved microbial-specific patterns^39^,^42^, shares NFκB as a common downstream effector and transcription factor, and exhibits TLR structural similarity^20^. TLR4 is uniquely critical to the regulation of innate immunity responses to gram-negative bacteria infection via binding of LPS, an outer membrane glycolipid of gram-negative bacteria. LPS is recruited by LBP (LPS-binding protein) and by CD14 to bind the TLR4-MD-2 complex^43^. Crystal structure analysis has revealed that TLR4 and its binding partner, MD-2, form a heterodimer that recognizes LPS present in gram-negative bacteria^20^ and structural analysis of TLR4-MD-2 interactions in the presence or the absence of the LPS antagonist, eritoran, indicates that MD-2 is essential for LPS binding to TLR4^20^.

Utilizing *in vitro* and *in vivo* approaches and *in silico* modeling analyses, we now demonstrate that NAMPT/PBEF directly induces TLR4-mediated NFκB activation without the requirement for MD-2-TLR4 binding and in the absence of additional LPS chaperones or cofactors. Multi-pronged *in silico* protein analysis revealed striking unconventional NAMPT/PBEF structural similarity with loop regions on MD-2 known to be involved in TLR4 binding and LPS-induced TLR4 activation. Both NAMPT/PBEF and MD-2 contain fully conserved K109, G110, E111 residues residing in these loops^20^ critical for TLR4 binding^20^,^23^. Furthermore, the residue R434 on NAMPT/PBEF exists in spatial proximity of the predicted loop binding residues and structurally aligns to R90 on MD-2, an amino acid that directly participates in TLR4-MD-2 interactions^27^. Together, these structural similarities in the TLR4 binding environment support the hypothesis that this loop functions as the TLR4-binding region for NAMPT/PBEF.

In contrast to the strong loop alignment between NAMPT/PBEF and MD-2 implicated in TLR4 binding, our model of signature binding surface of LPS-binding pockets^22^,^28^ determined that NAMPT/PBEF, unlike MD-2, fails to contain a well defined surface pocket for LPS binding. Furthermore, whereas MD-2 contains several highly clustered signature residues critical for LPS binding (F119, L74, L94, I52), these residues on NAMPT/PBEF are spatially separated and unlike MD-2, do not reside in close proximity to one another. Thus, NAMPT/PBEF is unlikely to directly and physically interact with either MD-2 or LPS within this region. Our analysis of the structure of the MD-2-LPS complex, however, indicates that LPS binding to TLR4 maps to the protruding region of NAMPT/PBEF (S402-N412), suggesting that NAMPT/PBEF may intrinsically adopt a conformation capable of direct TLR4 binding and activation.

In addition to NAMPT/PBEF and LPS/MD-2, Der-p2 and high-mobility group box 1 (HMGB1) proteins also bind TLR4. Similar to NAMPT/PBEF, HMGB1 is a ubiquitous dually functioning protein with intracellular activities as a DNA-binding protein regulating transcription^13^,^14^ as well as extracellular cytokine-like activities^12^,^15^ via TLR4 binding^11^,^12^. HMGB1 binds at least eight distinct receptors, including TLR4^4^,^8^,^13^ and is a key mediator of severe sepsis^44^ reproducing extracellular NAMPT/PBEF-mediated pathologic features (lung vascular permeability, interstitial edema, neutrophil infiltration) in rodent ARDS models^24^. HMGB1 levels in patients with sepsis and ARDS are highly correlated with morbidity and severity^24^,^32^,^35^ and HMGB1-TLR4 signaling has been implicated in the pathogenesis of sterile injury^26^. Unlike NAMPT/PBEF, however, HMGB1 does not directly bind TLR4^45^ utilizing a signaling cascade similar to LPS with high affinity binding to the MD-2/TLR4 complex^46^. In contrast to LPS and HMGB1, NAMPT/PBEF-mediated TLR4 binding and activation appears similar to Der-p2, a house mite allergen and TLR4-binding protein homologous to MD-2 (sequence identity 26.3%)^25^. The TLR4-binding region of Der-p2 is highly conserved and aligns to the NAMPT/PBEF loop^47^,^48^ and like NAMPT/PBEF, Der-p2 reconstitutes LPS-driven TLR4 signaling without the requirement for MD-2^20^,^25^.

Taken together with the recognition that *NAMPT* promoter polymorphisms confer ARDS/VILI susceptibility and influence ARDS severity and mortality^10^,^25^, these studies significantly increase the mechanistic understanding of the untoward inflammatory effects of mechanical ventilation-induced mechanical stress. Although NAMPT/PBEF is not entirely unique in its capacity to bind and activate TLR4, MD-2, HMGB1 and LPS fail to directly induce TLR4-mediated NFκB activation. Furthermore, unlike the house mite allergen, Der-p2, NAMPT/PBEF is endogenously expressed in man with critical participation in normal cellular homeostasis as well as in pathologic stress responses to excessive mechanical stress such as observed in the critical care setting. Thus, NAMPT/PBEF is unique in serving as an endogenous innate immunity molecule capable of directly binding and activating TLR4 in the absence of bacterial infection and cofactor requirements, thereby delineating a novel dimension to the induction of lung inflammatory and innate immunity responses by non-infectious mechanisms.

## Materials and Methods

### Reagents

Commercially available recombinant NAMPT/PBEF exhibits batch/lot variability on NFκB signaling, possibly via post-translational modifications or loss of bioactivity. Bioactive, non-denatured recombinant human NAMPT/PBEF and recombinant mouse NAMPT/PBEF was purchased from MBL International (Woburn, MA) and used in all *in vitro* and *in vivo* experiments, respectively. Individual lots were pre-screened and selected based on bioactivity/NFκB signaling induction. Other purchased reagents: recombinant human TLR4 and TNF-α (R&D Systems, Minneapolis, MN); lipopolysaccharide (Sigma, St. Louis, MO); immunoblotting antibodies (Cell Signal, Beverly, MA); immunostaining antibodies (BD Transduction Labs, San Jose, CA); immunohistochemistry antibodies (Sigma); SPR antibody (Bethyl Laboratories, Montgomery, TX); TLR4 neutralizing antibodies/inhibitors (Invivogen, San Diego, CA), Dual luciferase reporter assays (Qiagen, Valencia, CA). The neutralizing polyclonal NAMPT/PBEF antibody was generated as previously reported^12^.

### Cell Culture

Human pulmonary artery endothelial cells (EC) and human lung microvessel EC (Lonza, Walkersville, MD) were cultured as described previously^49^ in endothelial growth medium-2 (EGM-2 or EGM-2-MV). Cells were grown at 37 °C in a 5% CO_2_ incubator, and passages 6 to 9 were used for experiments. Media were changed one day before experimentation.

### Western Blotting

After treatment as outlined for individual experiments, EC were subsequently washed with cold (4 °C) Ca^2+^/Mg-free PBS and lysed with 0.3% SDS lysis buffer containing protease inhibitors (1 mM EDTA, 1 mM phenylmethylsulfonyl fluoride, 1 mM sodium orthovanadate, 1 mM sodium fluoride, 0.2 trypsin inhibitor unit/ml aprotinin, 10 μM leupeptin, and 5 μM pepstatin A). Sample proteins were separated with 4 to 15% SDS-PAGE gels (Bio-Rad, Hercules, CA) and transferred onto Immobilon-P polyvinylidene difluoride membranes (Millipore Corporation, Bedford, MA). Membranes were then immunoblotted with primary antibodies (1:1000, 4 °C, overnight) followed by secondary antibodies conjugated to horseradish peroxidase (1:5000, room temperature, 30 min) and detected with enhanced chemiluminescence (Pierce ECL or SuperSignal West Dura; Pierce Biotechnology, Rockford, IL) on Biomax MR film (Carestream Health, Rochester, NY). Western blot densitometry analysis was performed on inverted images with Adobe Photoshop (San Jose, CA) software, using the same selection box size for all band histogram means readings, where all means were then compared to the appropriate control on the same gel. The number of replicates analyzed is a minimum of three per experiment, with specific number of replicates and comparison control listed in each corresponding figure legend.

### Immunostaining

HLMVEC were cultured in EGM-2-MV culture medium in a 12-well plate format for immunostaining. The next day, the culture medium was changed and EC were incubated for up to six hours with either vehicle, TNF-α (100 ng/ml concentration), or rPBEF (10 μg/ml). Coverslips were dipped in D-PBS, immersed in 3.7% formaldehyde/PBS, pH 7.4, for 20 min at room temperature, washed and quenched in 50 mM NH_4_Cl/PBS 3 × 5 min, permeabilized in 0.1% TritonX100/PBS for 2-3 min, and blocked in 5% BSA/PBS for 60 min at room temperature. EC were incubated 60 min with NFκB monoclonal antibody (BD Transduction Labs) diluted 1:100 in blocking buffer, washed in PBS, incubated with goat anti-mouse IgG/AlexaFluor488 (Life Technologies, Carlsbad, CA) and subjected to autoradiography measurements. For the immunofluorescence experiments, EC were washed in PBS and mounted onto a drop of ProLong Gold with DAPI (Life Technologies). Images were acquired on a Leica TCS SP5 AOTF laser-scanning confocal microscope system scanning at 400 Hz with an Ar488 nm laser and a multiphoton 740 nm laser, a Leica DMI 6000 microscope, and an HCX PL APO CS 63X NA1.4 oil objective lens. Twelve-bit 512 × 512 images were acquired sequentially scan line-by-scan line and with a line average setting of 16 with Leica LAS AF software and detected with a photomultiplier tube. Images were analyzed using ImageJ v1.39 software (Wayne Rasband, National Institutes of Health, Bethesda MD).

### Surface Plasmon Resonance (SPR)

SPR was performed using a ProteOn XPR36 instrument and GLC sensor chips (Bio-Rad Laboratories, Hercules CA) and binding interactions simultaneously monitored after rPBEF (100 nM), rTLR4 (1 μM), and rPBEF-rTLR4 pre-mixed, individually flowed over PBEF antibody-coated surface (standard direct immobilization). Proteins were diluted in PBST buffer (10 mM phosphate, 150 mM NaCl, 0.05% Tween20 pH 7.4).

### Transgenic and Control Mice

All *in vivo* mouse methods/experiments were approved by and performed in accordance with University of Illinois at Chicago IACUC Committee guidelines and regulations. Animals were housed under standard conditions. NAMPT/PBEF^+/–^ mice were generated as previously described^12^ with C57BL/6 and TLR4-deficient (TLR4^−/−^) (*B6.B10ScN-Tlr4*^*lps-del*^*/JthJ*) mice (8–12 weeks old) purchased from Jackson Laboratory (Bar Harbor, ME).

### Recombinant PBEF-Induced Lung Injury and Ventilation-Induced Lung Injury (VILI) Models

rPBEF (40 μg/mouse) was given intra-tracheally (i.t.) for 4.5 hr and VILI was induced (40 ml/kg tidal volume, 4 hr) utilizing a rodent ventilator (Harvard Apparatus, Boston MA) as previously described^12^. Mice were pre-treated with RS (100 μg intra-peritoneally) 1 hr prior to rPBEF or VILI challenge.

### Bronchoalveolar Lavage (BAL) Analysis

Mice underwent lavage with 1 ml of HBSS buffer into the intratracheal catheter for BAL protein, total BAL cell counts and differential counts assessments as previously described^12^. Briefly, BAL fluid recovered was centrifuged, and the supernatant assessed for total protein content, using a kit assay (Bio-Rad) and expressed in mg/ml. In addition, the BAL pellet was utilized for counting the total number of cells with a TC20 cell counter (Bio-Rad), and for cytospin analysis on stained slides using Diff-Quik dye for differential counts (PMNs neutrophil percentage) from each mouse sample. BAL fluid and pellet were frozen for further analysis.

### Lung Tissue Histology

Excised mice left lungs were placed immediately in formalin overnight, followed by embedding in paraffin for histological evaluation by hematoxylin–eosin (H&E) staining. These sections were examined under microscope and representative images were recorded by camera^50^.

### Immunohistochemistry

Paraffin blocks of lung tissues were prepared and 10 μm microscope slides were obtained. A serial section of each specimen was de-paraffinized and rehydrated in serial graded ethanol. Antigens retrieval was achieved with 100  mM Tris base buffer (pH 9) and heating slides in a 98 °C water bath for 15  min. Endogenous peroxidase activity was blocked in methanol containing 3% hydrogen peroxide. The section was incubated with the p-NFκB (anti-phospho-RELA (p65/pSer^536^), Sigma) antibody produced in rabbit in 1:100 dilution for 40 min, followed by 30 min incubation with Dako labeled polymer-HPR anti-rabbit secondary antibody (K4011, Dako Inc, Carpinteria, CA). The DAB/DAB+ Chromogen solutions were used serially, and the slides were counterstained with hematoxylin.

### Microarray Analysis

RNA extraction was performed using RNeasy kits (Qiagen); Affymetrix Mouse Genome 430 2.0 and Mouse Gene 2.0 ST arrays used to detect genome-wide expression levels summarized by the gcrma package in Bioconductor with GC robust multichip average (GCRMA) normalization^51^. The expression level of each transcript in Mouse Gene 2.0 ST arrays was summarized by the RMA method in “oligo” package in Bioconductor^52^. SAM (Significance Analysis of Microarrays)^53^, implemented in the samr library of the R Statistical Package, for comparing log2-transformed gene expression levels between groups. Enriched BIOCARTA pathways were searched among differentially- expressed genes using NIH/DAVID^54^.

### *In Silico* Computational Modeling

*In silico* analysis are as previously described^22^,^28^. The structures of LPS binding pockets analyzed include: 1N12 (E. coli), 4GGM (caulobacter), 3MU3 (jungle fowl), 3RGY (cattle), 3VQ1 (mouse), 4G8A and 2E59 (human).

### Statistical Analysis

For all *in vitro* (n of 3 or more) or *in vivo* (n of 3-6) experiments, values are shown as the mean ± SEM and data were analyzed using standard student’s t test or two-way ANOVA. Significance in all cases was defined at *p* < 0.05.

## Additional Information

**How to cite this article**: Camp, S. M. *et al.* Unique Toll-Like Receptor 4 Activation by NAMPT/PBEF Induces NFκB Signaling and Inflammatory Lung Injury. *Sci. Rep.* **5**, 13135; doi: 10.1038/srep13135 (2015).

## Supplementary Material

Supplementary Information
