# Supplementary material for: Unique Toll-Like Receptor 4 Activation by NAMPT/PBEF Induces NFκB Signaling and Inflammatory Lung Injury
Source: Sci Rep. 2015 Aug 14;5:13135. doi: 10.1038/srep13135 (PMC4536637; doi:10.1038/srep13135)

Supplemental Data for:

Unique Toll-Like Receptor 4 Activation by NAMPT/PBEF Induces NFκB Signaling and Inflammatory Lung Injury

Sara M. Camp*[1], Ermelinda Ceco*[2], Carrie L. Evenoski*[1], Sergei M. Danilov[3], Tong Zhou[1], Eddie T. Chiang[1], Liliana Moreno-Vinasco[2], Brandon Mapes[2], Jieling Zhao[4], Gamze Gursoy[4], Mary E. Brown[2], Djanybek M. Adyshev[2], Shahid S. Siddiqui[2], Hector Quijada[1], Saad Sammani[2], Eleftheria Letsiou[2], Laleh Saadat[2], Mohammed Yousef[5], Ting Wang[1], Jie Liang[4], Joe G. N. Garcia[§1]

[1]Department of Medicine and Arizona Respiratory Center, The University of Arizona;
[2]Institute for Personalized Respiratory Medicine, University of Illinois at Chicago;
[3]Department of Anesthesiology, University of Illinois at Chicago
[4]Department of Bioengineering, University of Illinois at Chicago
[5]Life Science Group, Bio-Rad Laboratories, Inc.
*Contributed equally to this work

[§]**Corresponding Author:**
Joe G. N. Garcia, MD
Senior Vice President for Health Sciences
Arizona Health Sciences Center
University of Arizona
1295 N. Martin Ave., Tucson AZ 85721-0202
Phone: (520) 626-1197
Fax: (520) 626-1460
Email: skipgarcia@email.arizona.edu

Running Title: Toll-Like Receptor 4 Activation by NAMPT/PBEF

Supplement Figure 1. Isotype control immunohistochemistry staining of p-NFκB in lung tissues. Either SB (control, top) or VILI (bottom) mouse lung sections were stained with the same immunohistochemistry protocol/reagents as previously reported minus the selective p-NFκB primary antibody, which was replaced with a non-selective IgG control antibody. No clear positive staining were identified in any of these slides. Representative images shown. Scale bar = 100μM.

IHC Isotype Controls

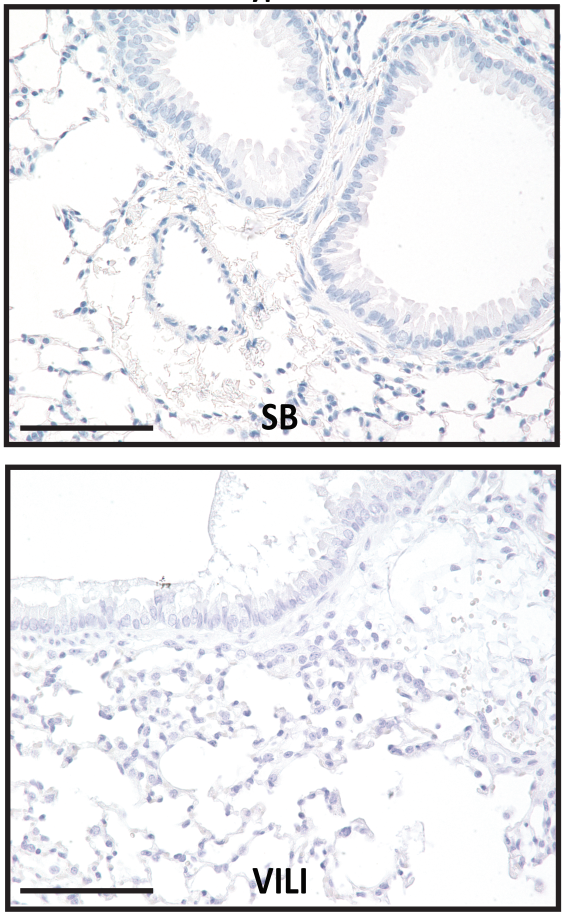

Supplement: Supplementary Information [file srep13135-s1.pdf]
